# Recent Advances and Promises in Nitrile Hydratase: From Mechanism to Industrial Applications

**DOI:** 10.3389/fbioe.2020.00352

**Published:** 2020-04-24

**Authors:** Zhongyi Cheng, Yuanyuan Xia, Zhemin Zhou

**Affiliations:** Key Laboratory of Industrial Biotechnology, Ministry of Education, School of Biotechnology, Jiangnan University, Wuxi, China

**Keywords:** Nitrile hydratase, natural distribution, gene types, catalytic mechanism, post-translational modification, thermostability, selectivity, industrial application

## Abstract

Nitrile hydratase (NHase, EC 4.2.1.84) is one type of metalloenzyme participating in the biotransformation of nitriles into amides. Given its catalytic specificity in amide production and eco-friendliness, NHase has overwhelmed its chemical counterpart during the past few decades. However, unclear catalytic mechanism, low thermostablity, and narrow substrate specificity limit the further application of NHase. During the past few years, numerous studies on the theoretical and industrial aspects of NHase have advanced the development of this green catalyst. This review critically focuses on NHase research from recent years, including the natural distribution, gene types, posttranslational modifications, expression, proposed catalytic mechanism, biochemical properties, and potential applications of NHase. The developments of NHase described here are not only useful for further application of NHase, but also beneficial for the development of the fields of biocatalysis and biotransformation.

## Introduction

Nitriles (RCN) are generally toxic, carcinogenic, mutagenic, and widespread organic compounds, that are primarily found in nature as cyanogenic glycosides, cyanolipids, β-cyanoalanine, and mandelonitrile (Legras et al., 1990). Plants, microbes, insects, and arthropods are found to be able to produce nitriles. The industrialization of nitriles production has led to their accumulation in ecosystems (Bhalla et al., 2012). In spite of their negative environmental impact, nitriles act as valuable intermediates in producing polymers, carboxylic acids, pharmaceuticals, and other fine chemicals (Martínková and Křen, 2010; Gong et al., 2012). The history of nitrile conversion follows the journey of this compound from its chemical hydration to its biotransformation. More than four decades ago, chemists Compagnon and Miocque pioneered the first general route of the chemical catalysis of nitriles to their corresponding carboxamides [RC(O)NH_2_] (Compagnon et al., 1968). However, the emergence of a biocatalyst for the synthesis of acrylamide ushered the nitrile hydration into a new era.

Nitrile hydratase (NHase) is one type of metalloenzyme that acts on the triple bond of a nitrile, catalyzing the substrate into amide product. The first discovery of such enzyme was reported in the bacterium *Rhodococcus rhodochrous* J1, which was identified as *Arthrobacter sp.* J1 at the very beginning (Asano et al., 1980) and then the focus of research shifted to the use of NHase in the biotransformation of nitriles to valuable amides (Chen et al., 2009). The biological route of amide production using NHase is superior to its chemical counterpart because the biocatalyst generally exhibits regio- and stereo-selectivity under mild reaction conditions, high catalytic efficiency, environment-friendly procedures and so forth. Yamada and Kobayashi (1996) initially managed to carry out the industrial-scale production of acrylamide using NHase from three generations of catalysts: *Rhodococcus* sp. N-774, *Pseudomonas chlororaphis* B23, and *R. rhodochrous* J1. Hitherto, the third-generation industrial strain *R. rhodochrous* J1 has dominated in producing industrial amide products, especially acrylamide, and nicotinamide. NHase is also found to have the capacity of improving the properties of polyacrylonitrile (PAN) fibers which act as important synthetic blocks in the textile industry (Tauber et al., 2000; Guebitz and Cavaco-Paulo, 2008). Beyond that, with the great achievements in recombinant DNA techniques, using engineered bacteria harboring robust NHase genes to produce amides and other industrial products is on the horizon.

Over the past few years, rapid progress with respect to NHase has been made and valuable information has been summarized. Quite a few reviews on the applications of NHase in biocatalysis have been published (Prasad and Bhalla, 2010), most of these focusing on either the industrialization of NHase or its structure and expression (Shen et al., 2012c; Mascharak, 2014; Gong et al., 2017; Supreetha et al., 2019; Jiao et al., 2020). In this review, we summarize newly available information regarding the natural distribution and types, posttranslational maturation, catalytic mechanisms, biochemical properties, and applications of NHase.

## Natural Distribution of NHase

### Prokaryotic NHase

Currently, bacteria are the major producers of NHase. In previous research, NHases were found in the genera Proteobacteria, Actinobacteria, Cyanobacteria, and Firmicutes (Foerstner et al., 2008). Conventional screening of NHase was still dominated by selective enrichment culture techniques using nitriles as the sole C/N source. *R. aff. qingshengii* was found to be able to degrade nitriles in Indonesia for the first time when grown in medium containing 100 mM acetonitrile (Hastuty et al., 2014). A bacterial strain, *Rhodococcus* sp. MTB5, was isolated from a soil sample contaminated by nitriles and was found to contain a NHase/amidase pathway which can produce metabolic intermediates such as benzamide and benzoic acid (Mukram et al., 2015). Most recently, by combining taxonomy and genome analysis approaches, several bacterial strains were isolated from the medium using acetonitrile as a nitrogen source and were found to be affiliated to different species (Egelkamp et al., 2017). However, such procedures using selective cultures were time-consuming and necessitated heavy workloads. Nowadays, gene mining has emerged as a useful approach for discovery of new enzymes and has been regarded as a promising alternative. Pei et al. (2014) successfully identified an NHase from *Pseudomonas putida* F1 by aligning the specific sequence motif of Fe-type NHase to all currently exist sequence databases.

All the aforementioned prokaryotic organisms have the capacity of using the nitrile-degrading pathway as a nutritional source for obtaining nitrogen and carbon on one hand and produce economically beneficial biotechnological products such as acrylamide, nicotinamide, and 5-cyanovaleramide on the other (Martínková, 2016). These organisms have gained extensive attention with respect to green catalysis and have shown great promises in fields of metabolic functions, mechanisms and phylogenetic distribution of NHase (Prasad and Bhalla, 2010).

### Eukaryotic NHase

Other than prokaryotes, eukaryotic organisms harboring NHase coding gene have been obtained since the first reported eukaryotic NHase in *Monosiga brevicollis*, a marine choanoflagellate, which is confidently placed within the opisthokonts (Foerstner et al., 2008). This eukaryotic NHase might have originated from a prokaryotic source by a gene transfer event (Nedelcu et al., 2008; Torruella et al., 2009; Sun et al., 2010). In another eukaryote, *Aureococcus anophagefferens*, a pelagophyte brown alga belonging to the stramenopiles, a gene that encodes the alpha subunit of NHase was found, and it shares higher similarity to the *M. brevicollis* NHase than to those prokaryotic NHases (Gobler et al., 2011). Marron et al. (2012) reported the presence of NHases in several eukaryotic supergroups including opisthokonts, amoebozoa, and archaeplastids. Most of these NHase genes are in β-α subunit fusion form, suggesting that all eukaryotic NHase may share a common ancestor harboring a gene coding subunit-fused NHase (Marron et al., 2012). The latest report on eukaryotic NHase was the isolation of two NHases from the Mediterranean sponge *Aplysina cavernicola*. The NHases were found to be able to specifically biotransform the cyano group of aeroplysinin-1 into amide group, and intriguingly, they might also catalyze the hydroxyl group elimination reaction of the substrate (Lipowicz et al., 2013). *De novo* sequencing showed no sequence homology between these two enzymes and other known NHases. The emerged eukaryotic NHases, and their unique gene types might elucidate further theoretical and biotechnological applications of this green catalyst.

## Types of NHase

The majority of characterized NHases coordinate either a non-heme low-spin trivalent iron ion or a non-corrinoid low-spin trivalent cobalt ion at their catalytic center. However, there are some exceptions: the NHase from *Rhodococcus jostii* was found to harbor three different metal ions (Co, Cu, Zn) (Okamoto and Eltis, 2007). In addition, two NHases from the Mediterranean sponge *A. cavernicola* seem not to strictly depend on iron or cobalt ions, they might bind manganese ions at the active center instead. The nickel ion could also recover their NHase activity (Lipowicz et al., 2013). Crystal structures have shown clear evidence that sulfur atoms of the three active site cysteine residues are coordinated to the metal ion in either Fe-NHase or Co-NHase, with two in an equatorial direction and the rest one in the axial direction. The metal ion is also coordinated with two nitrogen atoms from peptide backbone amides and bound to either a water molecule at the sixth coordination site of cobalt or one molecule of nitric oxide (NO) for Fe-type NHase (Yano et al., 2008). On the one hand, the iron or cobalt ion at the active site helps to improve the hydration process of substrate; on the other hand, the metal ions could also aid in the NHase folding (Banerjee et al., 2002).

### Fe-Type NHase

Typically, *Rhodococcus erythropolis* is the main organism producing Fe-type NHase, and NHases coordinated with iron ion at the active site are also discovered in organisms such as *P. chlororaphis*, *P. putida* or *Bacillus* sp. (Banerjee et al., 2002; Pei et al., 2014). These Fe-NHases usually share high amino acid sequence similarities with each other. Even the least similar NHases known until now showed approximately 62 and 57% identities in the α and β subunit, respectively, compared to the well-studied rhodococcal NHases (Pei et al., 2014).

Intriguingly, besides the iron ion, light is also of great necessity for the Fe-NHase activity. While aerobically incubated in dark environment, the Fe-type NHase shows dramatically low activity. However, illumination could recover such loss of catalytic activity. Spectroscopic studies demonstrated that the replacement of a NO molecule by water or hydroxide contributes to the recovery of Fe-NHase activity and changes the enzyme from the inactive form to active form (Noguchi et al., 1996; Odaka et al., 1997; Popescu et al., 2001).

### Co-type NHase

Compared with the Fe-NHase, the Co-type NHase has a higher frequency of occurrence to a certain extent (Liu et al., 2012). The most typical and well-known Co-type NHases are those from *R. rhodochrous*. The metal selectivity of such NHase was proved to be a *cblA*-dependent regulation process involving both NHase transcription and NHase maturation. The high complexity of the NHase maturation process enabled the strict selectivity of such NHase toward cobalt instead of other metals such as nickel (Lavrov et al., 2019). These *R. rhodochrous* NHases consist of four and up to 20 subunits and are divided into two subtypes according to their different molecular weight (low-molecular-weight and high-molecular-weight NHase). The high-molecular-weight NHase (H-NHase) seems to only occur in *R. rhodochrous*. Other organisms such as *Pseudonocardia thermophila* and *P. putida* also harbor Co-type NHase with low molecular weights (L-NHase). The H-NHase, which shows high thermal stability and excellent tolerance to organic solvent, has been industrially used in acrylamide and nicotinamide production (Mathew et al., 1988; Prasad et al., 2005). Unlike the Fe-type NHases, the Co-NHases show no photoregulation via NO binding and instead, the coordination site is replaced by a water molecule.

### Gene Structure of NHase

**FIGURE 1:**
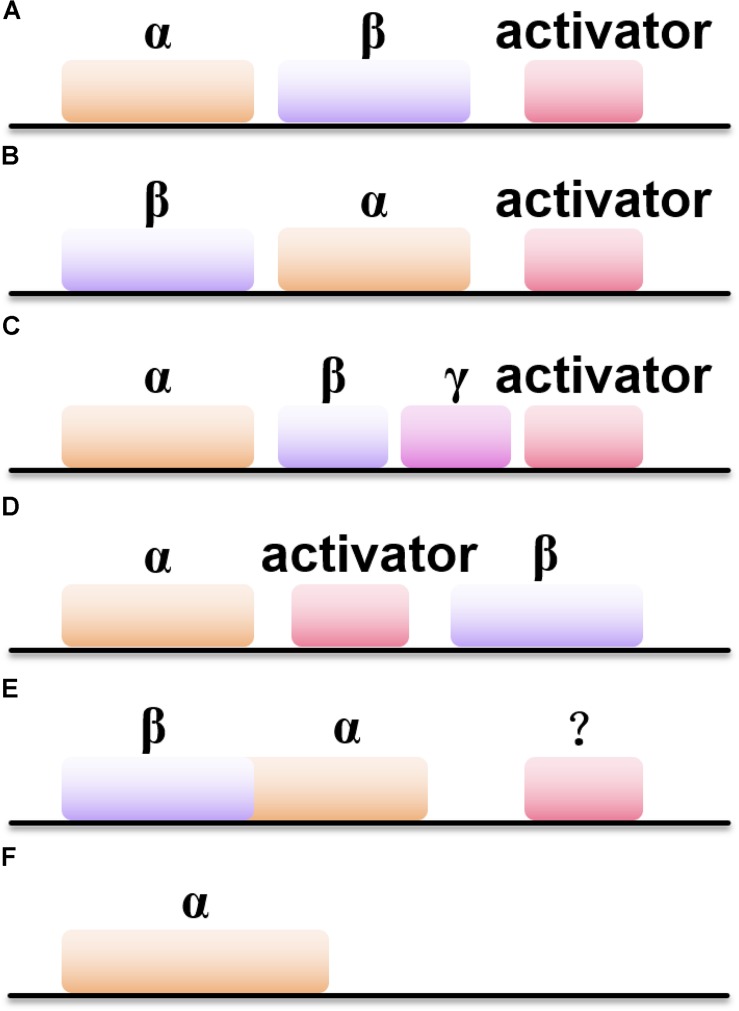
Six types of NHases with different gene organizations. **(A)** The gene order of < α-subunit> < β-subunit><activator>; **(B)** the gene order of < β-subunit> < α-subunit><activator>; **(C)** the gene order of < α-subunit> < β-subunit> < γ-subunit><activator>; **(D)** the gene order of < α-subunit><activator> < β-subunit>; **(E)** the gene order of < β-subunit> < α-subunit>; **(F)** the gene order of solo-subunit NHase.

Besides the difference in metal ions coordinated inside the active site of NHases, the gene structures of NHases also vary (Figure 1). Most of the Co-type NHases show the gene order of < β-subunit> < α-subunit><activator> (Figure 1B), while Fe-type NHases usually exhibit the order of < α-subunit> < β-subunit><activator> (Figure 1A; Guo et al., 2015). Interestingly, the thiocyanate-degrading enzyme (SCNase) from *Thiobacillus thioparus* has the gene order of < α-subunit> < β-subunit><γ-subunit><activator> (Figure 1C), whereas ANHase from *R. jostii* RHA1 exhibits the order of < α-subunit><AnhE> < β-subunit> (Figure 1D; Arakawa et al., 2007). *M. brevicollis* and several other eukaryotic sources host subunit fused NHases (α- and β-subunits are linked by a natural (His)_17_ linker) with the gene order of < βα-subunit> (Figure 1E), in which their activators remain undiscovered. Intriguingly, the (His)_17_ region which links the α- and β-subunits is not necessary for metal ion incorporatin of NHase (Yang X. et al., 2018). In addition, two novel solo-subunit NHases were reported recently in *Rhodococcus aff. Qingshengii* and alphaproteobacteria strain THI201 (Figure 1F; Hussain et al., 2013).

## Posttranslational Maturation of NHase

### Properties and Function of NHase Activator

In NHase, it turns out that the metal centers play important roles in stabilizing the enzyme structure and make great contributions to catalysis. However, if the transportation of metal ions is unregulated, the concentration of metal ions inside cells will be either too high or too low, which could result in severe cell damage or loss of protein function (Holm et al., 1996). A set of accessory proteins, termed as activators, has been found to assist NHase with the specific incorporation of certain metal ions. Activator genes have been found in almost all NHase gene clusters and they turn out to be indispensable for full catalytic activity (except for those eukaryotic NHases in which no gene referring to activator has been found to date) (Marron et al., 2012). Interestingly, the amino acid sequence and molecular mass of the activators of Fe- NHase and Co-type NHase differ a lot whereas the α and β subunits of both Fe-NHase and Co-type NHase share high sequence similarity, suggesting that the mechanism of metallocenter assembly is probably different (Cameron et al., 2005; Zhou et al., 2010). The evidence for the specificity of activators for different metal ions was observed by metal substation approach: Fe-type NHase from *Rhodococcus* sp. N-771 could incorporate cobalt ion but only showed 5.9% of wild-type activity (Nojiri et al., 2000). Miyanaga et al. (2004) attempted to replace the iron with cobalt ion inside Co-type NHase from *P. thermophila*, but obtained extremely low activity. Pei et al. (2017) proved that the activator from *Aurantimonas manganoxydans* ATCC BAA-1229 could be replaced by Co-type activator from *Pseudomonas thermophilus* JCM3095 and *P. putida* 5B to aid in the functional expression of its corresponding NHase, but not by that of the Fe-type NHase from *P. putida* F1.

In addition to facilitating iron or cobalt ion insertion, activators might also be responsible for cysteine oxidation at the active site (Zhou et al., 2009). By incubation with an oxidant, the activator could help NHase incorporate an iron ion and activate the enzyme by oxidation of its iron center (Nojiri et al., 2000). It was further demonstrated that the activator of L-NHase from *R. rhodochrous* J1 was involved in the active site Cys oxidation *in vitro* (Fang S. et al., 2015). In addition, it is reported that the flexible C-terminus domain of one Co-type activator, P14K (activator from *P. putida* NRRL-18668), is positively charged and might help cobalt-free NHase overcome energy barriers, leading to the formation of a cobalt-containing NHase (Liu et al., 2014a).

### Self-Subunit Swapping Chaperones

**FIGURE 2:**
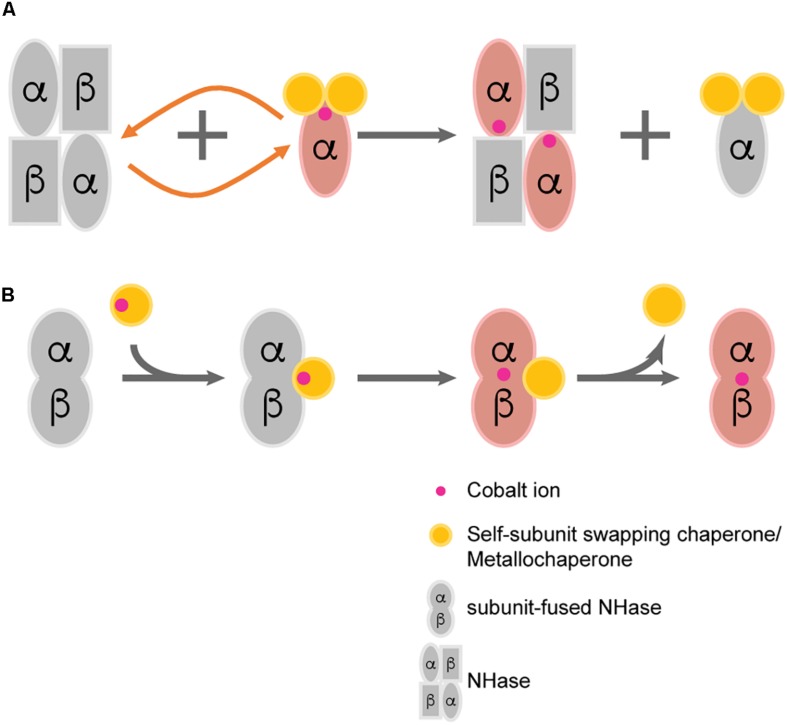
Two proposed post-translational modification patterns of NHase. **(A)** Self-subunit swapping; **(B)** Direct metal ion incorporation regulated by metallochaperone.

A novel hypothesis named “self-subunit swapping” was stated years ago regarding cobalt uptake in *R. rhodochrous* J1 (Zhou et al., 2008; Figure 2A). It is quite unique compared with those known mechanisms of metallo-center biosynthesis (Kuchar and Hausinger, 2004). By using Dynamic light scattering (DLS) and size exclusion chromatography, Zhou et al. (2010) found the formation of large complexes such as a proposed αe_2_ intermediate. The α-subunit from αe_2_ intermediate could swap with the cobalt-free α-subunit from apo-NHase. One would expect that there should be a driving force for the swapping of subunits. The driving force for such subunit swapping turns out to be dependent on the formation of salt bridges between two highly conserved arginine residues (R52 and R157) at the β-subunit and the two oxidized cysteine residues (Cys-112 and Cys-114) at the holo-α-subunit (Huang et al., 1997; Nagashima et al., 1998; Hourai et al., 2003). It was hypothesized that the electrostatic force required to form such salt bridges triggers α-subunit exchange by observing the failure of the α-subunit swapping between apo-αe_2_ and apo-L-NHases *in vitro* (the apo-α-subunit of apo-αe_2_ lacks the oxidized cysteine) (Zhou et al., 2008). According to these findings, the activator protein was termed as a subunit swapping chaperone. Moreover, the activator was also proposed to possess a redox function because of the occurrence of the active-site cysteines oxidation after cobalt insertion (Zhou et al., 2009). Apart from the first discovery of self-subunit swapping in the L-NHase of *R. rhodochrous* J1, such protein posttranslational modification was afterward found to exist in another Co-NHase from *P. putida* NRRL-18668 (Liu et al., 2012), and it is speculated to exist in various other Co-NHases including thiocyanate hydrolase, in which two cysteine residues are also posttranslationally modified and bound to a unique non-corrin cobalt center (Arakawa et al., 2007).

### Metallochaperone

Metallochaperones are known to deliver suitable metal ions to a certain protein, while the receptor protein has ligand specificity and generally has a higher affinity for metal ions (Rosenzweig, 2002). The metallochaperone role of Fe-NHase activator has already been clarified. Sequence alignment of the Fe-type NHase activator indicates that there is a conserved cysteine-rich motif (C-X-C-C) in all Fe-type activators, and this motif is found to act as a metal binding site in other metallochaperones (Lu et al., 2003; Cheng et al., 2013). Most recently, a Fe-type NHase activator protein from *Rhodococcus equi* TG328-2 was successfully obtained for the first time. This activator presents distinct GTPase motifs and exhibits GTPase activity. It was identified as a member of the COG0523 subfamily of G3E P-loop GTPases, a diverse group of GTPases with proposed roles in metal homeostasis (Gumataotao et al., 2016). These results also demonstrated that metal binding could regulate GTPase activity, and might inspire the further study on COG0523 protein function (Sydor et al., 2013).

Previously, the Co-type activators were proposed to facilitate self-subunit swapping of NHase for enzyme maturation. However, by fusing the α- and β-subunit as one single peptide, Zhou’s group successfully identified the metallochaperone function of the Co-type activator (Xia et al., 2018b). By incubating apo-fused-NHase with Co-type activator, significant increase in the specific activity of apo-fused-NHases was observed, indicating that the activator can transfer cobalt ions to apo-NHases. However, in such circumstance, self-subunit swapping was unlikely to occur because the separated subunits were fused by a flexible linker. There should be a direct transportation of cobalt ion from activator protein to apo-NHase without the formation of the previously described subunit swapping intermediate (such as αe_2_ from *R. rhodochrous* J1). It is highly possible that both the self-subunit swapping and direct cobalt transportation occur in the biosynthetic process of Co-type NHase, although which pattern is in the dominant place remains unknown (Figure 2B).

## Expression of NHase

As mentioned earlier, many microbial genus have been isolated, exhibiting NHase activity (Wang et al., 2000; Okamoto and Eltis, 2007; Raj et al., 2007; Zheng et al., 2008). Among these, several wild strains have been selected for industrial biosynthesis of amide products (Watanabe et al., 1987; Nagasawa et al., 1989, 1993; Wang et al., 2007). However, some of these wild strains inevitably contain amidase or nitrilase, which could result in the accumulation of carboxylic acids and thus lead to the impurity of the product. With the rapid development of cloning techniques, great progress has been made in the field of heterologous expression of NHase, which make it possible for the production of pure amide products. Until now, *Escherichia coli* (Pei et al., 2014; Lan et al., 2017; Wang et al., 2017), *Pichia pastoris* (Wu et al., 1999; Shi et al., 2004; Pratush et al., 2017), *R. rhodochrous* (Mizunashi et al., 1998), *Bacillus sp.* (Singh et al., 2018), and *Corynebacterium glutamicum* (Yang et al., 2019a) have been selected for the heterologous expression of NHases (Table 1).

**TABLE 1:** Summary of the advances in expression and purification of NHase in recent 5 years.

**Origin**	**Expression host and (plasmid)**	**Optimization of expression and purification approach**	**substrate**	**Catalytic ability**	**References**
*Pseudomonas putida* F1	*E. Coli*BL21(DE3) (pCDFDuet-1)	N-terminal 6 × His tag	3-Cyanopyridine Acrylonitrile Isobutyronitrile 4-Chlorobutyronitrile Valeronitrile 4-Cyanopyridine Benzonitrile	26 ± 1.1 U/mg 941 ± 35 U/mg 775 ± 32 U/mg 553 ± 24 U/mg 535 ± 23 U/mg 37 ± 1.6 U/mg 22 ± 1.0 U/mg	Pei et al., 2014
*Rhodococcus ruber* TH3	*Rhodococcus ruber* TH3 (pNV18.1)	Co-expression with *gro*EL-ES	acrylonitrile	4342 U/mL	Tian et al., 2016
*Rhodococcus rhodochrous* J1	*E. Coli*BL21(DE3) (pET-24a)	Codon optimization/RBS engineering/Strep tag	3-cyanopyridine	400 U/mg (L-NHase) 234 U/mg (H-NHase)	Lan et al., 2017
*Rhodococcus rhodochrous* PA-34 mutant 4D	*Pichia pastoris* KM-71 (pHIL-D2)	Not available	3-cyanopyridine	5.5 U/mg DCW	Pratush et al., 2017
*Pseudoxanthomonas*sp. AAP-7	*E. Coli*BL21(DE3) (pET-29a (+)/pET-21a (+)/pGEX-4t-1)	Co-expression with *gro*EL-ES/C-terminal 6 × His tag	3-cyanopyridine	33.7 ± 2.6 U/mg	Zhang et al., 2017
*Bacillus* sp. APB-6	*Bacillus* sp. APB-6	Not available	acrylonitrile	100% bioconversion	Singh et al., 2018
*Rhodococcus ruber*TH	*E. Coli*BL21(DE3) (pET-28a)	Co-expression with novel chaperone *gro*EL2	acrylonitrile	202.8 U/mL	Chen et al., 2018
*Aurantimonas manganoxydans*ATCC BAA-1229	*Corynebacterium glutamicum* (pXMJ19/pEKEX2/pEC-XK99E)	promoter engineering/codon optimization/RBS engineering/Construction of mmp-based expression system	3-cyanopyridine	14.97 U/mg DCW	Yang et al., 2019a

To overcome the obstacle that there might be a low expression level of heterologous protein, as well as to avoid the formation of inactive and insoluble protein, tremendous efforts have been made including ribosome binding site engineering, molecular chaperone co-expression, and tag fusion strategy. Tian et al. (2016) introduced a shuttle plasmid which carries *ecgro*EL-ES, a molecular chaperone gene, to *R. ruber* TH3G strain hosting an NHase gene. The chaperones assist protein-folding and even reactivate the native NHases (Tian et al., 2016). Moreover, in *E. coli*, the soluble expression level of recombinant NHase could be improved with the help of molecular chaperones and thus low inducing temperature was avoided. The catalytic activity of NHase could be tremendously increased by coexpression with chaperones such as GroEL/ES and DnaK/J-GrpE as well (Pei et al., 2015). By co-expressing with putative activator P46K on two separate plasmids in *E. coli*, NHase from *Pseudoxanthomonas* sp. AAP-7 exhibited the maximal enzyme activity. In addition, the presence of chaperones GroEL-GroES substantially increased the solubility of the recombinant NHase (Zhang et al., 2017). Through transcriptome analyses, Chen et al. (2018) discovered novel chaperones GroEL2 and GroES from *R. ruber.* The chimeric NHase-GroEL2 could enhance both the stability and activity of NHase and meanwhile well balance the stability-activity trade-offs (Chen et al., 2018). By combining codon optimization, ribosome binding site (RBS) and spacer sequence engineering together, Zhou’s group successfully overexpressed the NHase from the industrial strain *R. rhodochrous* J1 in *E. coli* (Table 1). The specific activities of the H-NHase and L-NHase reached 234 U/mg and 400 U/mg, respectively (Lan et al., 2017).

As activators play important roles in the NHase maturation process, it is of great necessity that one can obtain large amounts of highly pure NHase activator so that the characteristic of activator could be fully studied. Nonetheless, some activators are expressed at an extremely low level, even undetectable in SDS-PAGE (Wu et al., 1997; Cameron et al., 2005; Petrillo et al., 2005). Liu found that the expression of NHase activator was related to the N-end rule, which indicated that the stability of a certain protein might be affected by its N-terminus residues (Varshavsky, 1997; Dougan et al., 2010). For example, proteins with N-terminus lysine shows a short half-life whereas substituting glycine for lysine could elongate the half-life of proteins (Tobias et al., 1991). Based on the N-end rule, P14K was successfully expressed in a higher level through molecular modification at its N-terminus (Liu et al., 2013). Most recently, by fusing SKIK tags to the N-terminus of the activator from *A. manganoxydans* ATCC BAA-1229, the expression level the activator along with the activity of the NHase were improved (Yang Z. et al., 2018).

## Catalytic Mechanism of NHase

**FIGURE 3:**
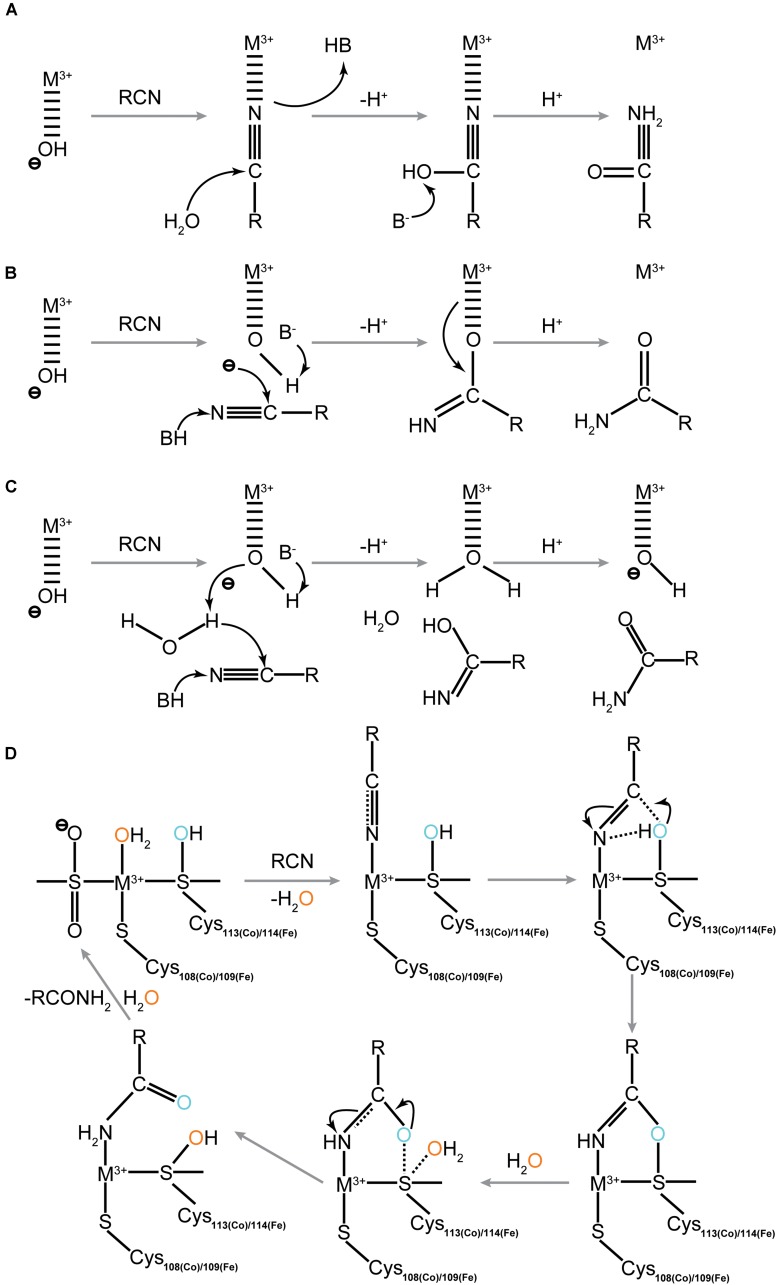
Four proposed catalytic mechanisms for NHase catalysis. **(A)** The innersphere mechanism; **(B)** the outer-sphere mechanism; **(C)** the newly proposed outer-sphere mechanism (Indirectly Activated Nucleophile); **(D)** Direct attack of activated Sulfenate toward substrate. The blue and orange color of the oxygen atoms represent the different sources of oxygen during catalytic process.

The catalytic mechanism of NHase has not yet been fully clarified. Quite a few studies on the model complexes of the NHase active site have been reported in recent years. Transitional metals such as Fe, Co, Pt, Rh, Ru, and Ni were used to construct artificial active centers of NHase to study the mechanism of NHase catalysis. The Oxidation states of the metal ions and cysteine residues at the NHase active site now represent intriguing research hotspots. Studies show that the oxidation states of the metal ions and cysteine residues at the NHase active site could kinetically influence the activity of NHase (Crisóstomo et al., 2010; Swartz et al., 2011; Li et al., 2012; Chang et al., 2015; Fang Y.-X. et al., 2015; Kumar et al., 2015; Yano et al., 2015). Based on these findings, together with the data referring to how the molecular structure controls enzyme function, several possible catalytic mechanisms of NHase have been proposed including the three conventional hypotheses (Figures 3A–C). The most convincing mechanism among these three involves the binding of the nitrogen atom of the nitrile substrate to the metal center (Hopmann et al., 2007). The nitrile group replaces the hydroxide/water which initially binds to the metal ion, enabling the base-activated water molecule to undergo nucleophilic attack. Three strictly conserved residues, βTyr72, αSer113, or αCys-SOH were proposed to be the potential base candidates for the nucleophilic attack (Mitra and Holz, 2007; Hopmann and Himo, 2008; Yamanaka et al., 2010), and the active site metal ion is probably coordinated with the nitrogen atom of the cyanide group (Hopmann, 2014; Martinez et al., 2014; Yamanaka et al., 2015). It is proposed that the carbon atom of the substrate cyanide group is attacked during catalysis, and such nucleophilic attack exhibited a lower energy barrier than the nucleophilic attack by the water molecule (Hopmann, 2014). Another proof suggesting that it is the nitrogen atom of the cyanide group coordinated with the metal ion at the sixth coordination site was presented by Yamanaka et al. (2015) through time-resolved crystallization. It is reported that the coordinated nitrile substrate interacts with βArg56 and αCys114 through hydrogen bonds (Hopmann, 2014). The importance of Arg56 in hydrogen bonding has been identified by site-directed mutations. In this situation, the corresponding βR56Y and βR56E variants completely lost their catalytic activities (Piersma et al., 2000). It is proposed that the nucleophilic attack of a water molecule to the sulfur atom of the αCys114 triggers the release of the amide product. Once the amide is released, the active site undergoes regeneration and is ready for the next nitrile substrate (Hopmann, 2014). The regeneration of the NHase active site firstly takes place once the amide product which is coordinated to the sixth coordination site of the metal ion was replaced by one water molecule. Based on the findings of Hopmann, the Shigeta group studied the initial steps of the catalytic mechanism of Fe-containing NHase using the QM/MM method, and they found that the cyclic intermediate formation path was the most probable reaction mechanism of NHase. In this mechanism, the substrate is directly attacked by αCys114-SO^–^ (Kayanuma et al., 2015). The same group then further examined the catalytic mechanism of Fe-containing NHase after the formation of the cyclic intermediate and proposed another reaction path differed from that suggested by Hopmann. Instead of the disulfide intermediate formed from the cyclic intermediate, the Shigeta group suggested that a direct attack of the water molecule on the sulfur atom of αCys114, which resulted in the formation of the imidic acid intermediate (Kayanuma et al., 2016).

The LS state plays an important role in the binding and activation of nitrile substrates and to maintain the LS state, oxidized thiolate ligands are very crucial (Martinez et al., 2014). A recent report further proved that the posttranslational sulfonate acts as the nucleophile that initially attacks the nitrile, and found the source of the product carboxamide oxygen is from the protein (Figure 3D; Nelp et al., 2016). Further quantum mechanics investigation identified that the nitrile hydration reaction evolves toward the product in only three steps. The hydroxyl group of the oxidized αCys113 (Co-type NHase)/αCys114 (Fe-type NHase) residue firstly acts on the carbon atom of the substrate and then leads to the amide formation which followed by the enzyme restoration phase (Prejano et al., 2017).

It is well-known that the active site of NHase contains a highly conservative catalytic motif (C-X-X-C-S-C) (Nagashima et al., 1998; Murakami et al., 2000; Noguchi et al., 2003). The serine in this motif is proposed to participate in the proton transfer pathway (Murakami et al., 2000), which contributes to the NHase maturation (Mitra and Holz, 2007; Yamanaka et al., 2010). These speculations indicated that the serine was crucial for NHase maturation. However, according to a recent study, the αSer115 in NHase from *Bordetella petrii* was found to be unnecessary for NHase catalysis. Quantum chemical calculation results showed that the energy of the proton transfer without the participation of αSer115 (type II) (−42.5 kcal/mol) was lower than that of the proton transferred to -NH2 via αSer115 (type I) (−13.2 kcal/mol); the corresponding S115A mutant retained nearly 40% of the original catalytic activity, indicating that αSer115 might facilitate the reaction though not indispensable (Sun et al., 2016a). Besides the conservative catalytic motif of NHase, three residues in Fe-type NHase, αR157, αH80, and αH81, were found to be catalytically important, but not essential. Mutagenesis on these sites resulted in dramatic decrease of *k*_cat_ values. Further structural study indicated that disruption of the hydrogen bonding interactions in mutant enzyme probably altered the nucleophilicity of the sulfenic acid oxygen and the Lewis acidity of the active site Fe (III) ion (Martinez et al., 2015).

## Biochemical Properties of NHase

### Regioselectivity

Asymmetric catalysis is the hottest and most cutting-edge research field in modern organic chemistry, and using chemical catalysts or enzymes to perform asymmetric catalysis has become one of the hottest research areas in recent years (Reetz, 2011). In terms of the biocatalysis of NHases, regioselectivity has emerged as a vital catalytic property and NHase has overwhelmed its chemical counterpart with respect to the hydration of various dinitrile substrates. Since NHase exhibits high regioselectivity during the aliphatic or aromatic dinitrile catalysis process, it has great potential to replace traditional chemical hydration of dinitriles, which is virtually impossible to show regioselectivity (Table 2; Meth-Cohn and Wang, 1997; Dadd et al., 2001; Li et al., 2001). One of the most representative examples is the enzymatic production of 5-cyanovaleramide using NHase, which demonstrates higher conversion, higher selectivity, and fewer by-products (DiCosimo et al., 1998; Hann et al., 1999; Shen et al., 2012b; Chen et al., 2013a). In addition, an NHase isolated from *Rhodococcus aetherivorans* ZJB1208, was reported to possess the ability to regioselectively biotransform 1-cyanocyclohexaneacetonitrile into 1-cyanocyclohexaneacetamide with a biocatalyst yield (g_product_/g_cat_) of 204.2. This result turns out to be a typical example of NHase showing both excellent regioselectivity and strong substrate tolerance for nitrile substrates and casts light on the potential industrial production of gabapentin (Zheng et al., 2016).

**TABLE 2:** Regio- and Stereo-selective NHase reported in recent years.

**Origin and (Potential application)**	**Substrate**	**Regio-/stereo-selectivity**	**Conversion rate**	**Main product**	**References**
*Pseudomonas putida* NRRL-18668 (Bioproduction of pharmaceuticals and agricultural chemicals)	Adiponitrile/malononitrile	Regioselectivity	99.5% (adiponitrile)	5-cyanovaleramide (95.7%) cyanoacetamide (97.8%)	Cheng et al., 2016
*Pseudomonas putida* NRRL-18668 βL37Y mutant (Bioproduction of pharmaceuticals)	Adiponitrile/malononitrile	Regioselectivity	98.8% (adiponitrile)	Adipoamide (96.1%) Malomamide (97.2%)	Cheng et al., 2016
*Comamonas testosteroni* 5-MGAM-4D (Bioproduction of pharmaceuticals)	Adiponitrile/malononitrile	Regioselectivity	100% (adiponitrile)	Adipoamide (97.9%) Malomamide (98.2%)	Cheng et al., 2016
*Comamonas testosteroni* 5-MGAM-4D βF37P mutant (Bioproduction of pharmaceuticals and agricultural chemicals)	Adiponitrile/malononitrile	Regioselectivity	94.1% (adiponitrile)	5-cyanovaleramide (90.4%) cyanoacetamide (96.6%)	Cheng et al., 2016
*R. aetherivorans* ZJB1208 (Bioproduction of pharmaceuticals and agricultural chemicals)	1-cyanocyclohexaneacetonitrile	Regioselectivity	100%	1-cyanocyclohexaneacetamide (966.7g/L)	Zheng et al., 2016
*Rhodococcus ruber* CGMCC3090 (Bioproduction of agricultural chemicals)	Adiponitrile	Regioselectivity	100%	5-cyanovaleramide (99.2%)	Shen et al., 2012b
*Rhodococcus rhodochrous* J1 (Bioproduction of pharmaceuticals)	Adiponitrile/malononitrile/terephthalonitrile/phthalodinitrile	Regioselectivity	98.6% (adiponitrile) 97.3% (malononitrile) 99.2%(terephthalonitrile) 96.1%(phthalodinitrile)	Adipamide (100%) Malomamide (77.3%) Terephthalamide (84.3%) Phthalamide (100%)	Cheng et al., 2018a
*Rhodococcus rhodochrous* J1 βY68T/W72Y mutant (Bioproduction of pharmaceuticals and agricultural chemicals)	Adiponitrile/malononitrile/terephthalonitrile/phthalodinitrile	Regioselectivity	70.5% (adiponitrile) 79.5% (malononitrile) 71.1% (terephthalonitrile) 72.5% (phthalodinitrile)	5-cyanovaleramide (100%) cyanoacetamide (97.1%) 4-cyanobenzamide (98.2%) 2-cyanobenzamide (100%)	Cheng et al., 2018a
*Rhodopseudomonas palustris*HaA2 (Bioproduction of pharmaceuticals)	2-phenylpropionitrile/2-Phenylbutyronitrile	Stereoselectivity	Not available	*S*-product (*E*-value > 100)/ *S*-product (*E*-value 53)	van Pelt et al., 2011
*Rhodopseudomonas palustris*CGA009 (Bioproduction of pharmaceuticals)	2-phenylpropionitrile/2-Phenylbutyronitrile	Stereoselectivity	Not available	*S*-product (*E*-value > 100)/ *S*-product (*E*-value 95)	van Pelt et al., 2011
*Rhodococcus* sp. AJ270 (Bioproduction of pharmaceuticals)	2-phenylbutyronitrile/3-Benzoyloxypentanedinitrile/Naproxennitrile	Stereoselectivity	Not available	*R*-product (*ee* 83%)/ *S*-product (*ee* 68.2%)/ *S*-product (*E*-value 80)	Wang, 2005; Song et al., 2007; van Pelt et al., 2011
*Rhodococcus rhodochrous* J1 (Bioproduction of pharmaceuticals)	*rac*-mandelonitrile	Stereoselectivity	87.3%	*S*-product (*ee* 52.6%)	Cheng et al., 2018b
*Rhodococcus rhodochrous* J1 βF37H mutant (Bioproduction of pharmaceuticals)	*rac*-mandelonitrile	Stereoselectivity	80.1%	*S*-product (*ee* 96.8%)	Cheng et al., 2018b

Although several bacterial strains exhibiting promising regioselectivity have been isolated, the fundamental question of why these strains show particular regioselectivity toward various nitrile substrates remains to be explained. It was not until recently that Zhou’s group discovered several clues into this intriguing issue. By sequence alignment approach, a substrate tunnel residue, βPhe37, was found to act as a switch for directing the regioselectivity of NHases. The enzyme regioselectivity toward several aliphatic α, ω-dinitriles was inverted followed by site-directed mutagenesis on this site. Further tunnel calculation and analysis (Petřek et al., 2006) indicated that the geometry of the substrate tunnel might be affected by the tunnel-forming amino acids, and thus the reaction regioselectivity was altered (Cheng et al., 2016). Beyond that, the substrate binding pocket residues were also investigated and engineered. Combining molecular docking approach with iterative saturation mutagenesis, a tailored NHase mutant, Y68T/W72Y was successfully screened out and showed a completely inverted regioselectivity toward dinitriles (Cheng et al., 2018a). All of these findings clarified the role of tunnel forming residues and substrate binding pocket residues on NHase regioselectivity toward dinitrile substrates and may shed light on the future modification of NHase regioselectivity toward other substrates with more than two nitrile groups.

### Stereoselectivity

One of the most prominent features of biotransformation is enantioselectivity. Due to the inherent enantiomeric properties of proteins, NHases act as enantioselective biocatalysts (Wang, 2015). During the past few years, studies focusing on stereoselective NHase have advanced tremendously (Table 2). Several stereoselective NHases from the genera of *Agrobacterium, Moraxella, Serratia, Rhodococcus*, and *Pseudomonas* have been isolated (Shen et al., 2012a). Pawar and Yadav (2014) cross linked NHase from *R. rhodochrous* ATCC BAA-870 with poly (vinyl alcohol) (PVA)/chitosan-glutaraldehyde and the immobilized NHase showed 81% enantiomeric excess (*ee*_p_) toward R-mandelonitrile. In addition, a bienzymatic NHase/amidase system synthesizing 1,2-disubstituted nitrile ferrocene derivative was reported recently. Whole cells of *R. rhodochrous* PA-34 harboring NHase could catalyze the biotransformation of nitrile ferrocene derivative into its corresponding amide with high enantioselectivity (D’Antona et al., 2016).

**FIGURE 4:**
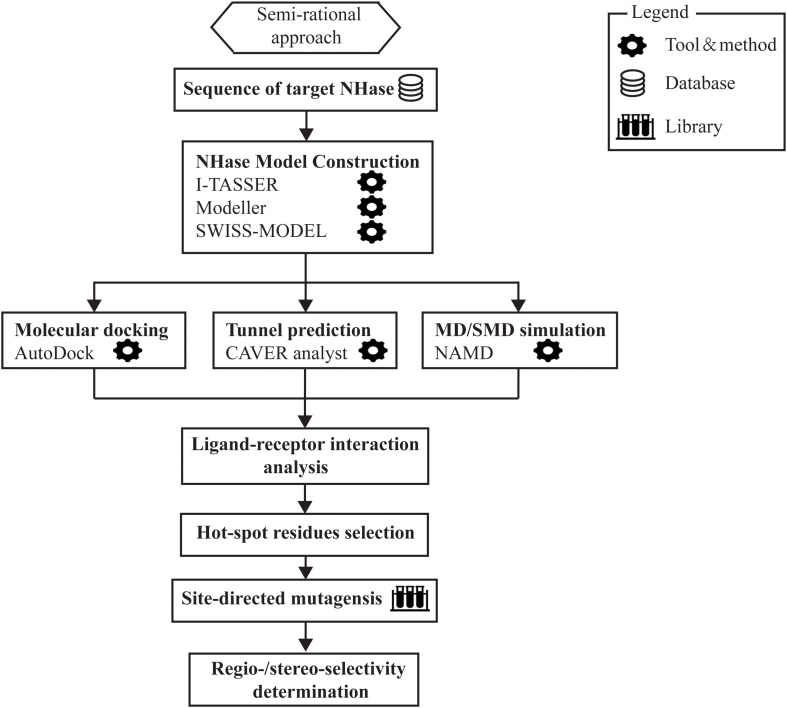
The outlay of semi-rational approach involved in the modification of regio- and stereo-selectivity of NHase.

Notwithstanding the existence of stereoselective NHases in nature, engineered NHases with higher stereoselectivity are highly desired but rare. Besides, the reason why NHases show intrinsic stereoselectivity needs to be explained. Experimental X-ray data and molecular dynamics simulation (MD) have demonstrated that some tunnel forming amino acids might influence the stereoselectivity of NHase (Peplowski et al., 2008). Recently, Cheng et al. (2018b) reported a new (semi-rational) approach to the development of stereoselective NHases with application to the resolution of racemic mandelonitrile. By combining molecular docking, substrate tunnel calculations and steered molecular dynamics (SMD) simulations, the authors successfully positioned the βPhe37 residue at the β subunit of the L-NHase from *R. rhodochrous* J1 which exhibited a 96.8% *ee*_p_ toward *S*-mandelonitrile after site-directed mutagenesis. The pronounced *S*-selectivity was then rationalized by modeling. These results may explain the fundamental question of how binding pocket or substrate access tunnel residues affect substrate orientation and thereby control the enzyme stereoselectivity, which would point out the direction for modulating biocatalysis stereoselectivity in the future (Cheng et al., 2018b). Most recently, by a comprehensive exploration of the substrate parameters for the L-NHase, researchers found that large and rigid substrates with steric hindrance are difficult to be accommodated by the enzyme’s catalytic site or the channel leading to it. Also, the electrophilicity of the nitrile might slow down the hydration reaction (Mashweu et al., 2020). Such results further emphasized the importance of the active site as well as the enzyme channel in determining the substrate profile and the enzyme selectivity, and through proper semi-rational approaches, well-tailored NHase mutants with excellent regio- or stereo-selectivity might be obtained (Figure 4).

### Stability of NHase

Nitrile hydratase plays an extremely important role in the industrial production of high purity amide products. However, most of the highly active NHase exhibit poor stability, which limits its wider application. For instance, the NHases from *P. chlororaphis* B23 and *Rhodococcus* sp. N-774 become unstable above 20°C (Hann et al., 1999; Ryuno and Nakamura, 2003), and the NHase from *R. rhodochrous* J1 is merely stable between 10 and 30°C (Wieser et al., 1998). Since the hydration of nitrile is an exothermic reaction, NHases must be stabilized by keeping the temperature at a low level, but this might result in an uncontrollable increase in energy cost. In addition, high concentrations of amide produced during the industrial process also call for NHase with high organic solvent tolerance. Therefore, a more robust NHase with high activity and high tolerance is extremely urgent for industrial amide production.

Thermal stability studies on NHases have been conducted for quite a long time. Liu et al. (2008) analyzed the crystal structures of thermophilic *Bacillus smithii* (PDB code: 1V29) and *P. thermophila* (PDB code: 1UGQ) and found that inter-subunit salt bridges between Asp, Arg and Glu residues are able to make the entire protein more rigid and thus enhance the NHase thermostability. One of the suggested regions involved in the thermostability of NHase (B1) in the β subunit in Liu’s study was used to engineer the NHase from *Rhodococcus ruber* TH (Chen et al., 2013b). Several charged amino acids such as Asp and Lys were introduced to the β subunit of the *R. ruber* TH NHase as well as its C terminus. Salt bridges formed between these newly introduced residues contributed tremendously to the enhanced thermostability of NHase from *R. ruber* TH.

The thermostability of NHase was also improved by protein fragment swapping (Table 3). Several fragments of NHae from *P. putida* which were vulnerable to high temperature were replaced by thermophilic fragments from *C. testosteroni* 5-MGAM-4D and *P. thermophila* JCM3095. The resulted chimeric NHases higher thermostability compared with their parent enzyme (Cui et al., 2014). In addition, Sun et al. (2016b) constructed a chimeric NHase by swapping the C-domains of NHase from *B. petrii* with that of the relatively thermo-stable NHase from *P. thermophile*, and the melting temperature (*T*_m_) of the chimeric enzyme increased by 5°C compared with that of the original NHase. Moreover, a thermophilic fragment of NHase from *P. thermophila* JCM3095 was select to substitute for the β-6th helix in NHase1229. The tailored enzyme showed improved thermostability and it is proposed that the β-6th helix might have an impact on the thermostability of NHase (Pei et al., 2018). These results suggested that the creation of chimeric proteins via fragment swapping strategy with low tradeoffs in activity may be an alternative to improve the stability of other enzymes.

**TABLE 3:** Summary of the advances in thermostability engineering of NHase in recent years.

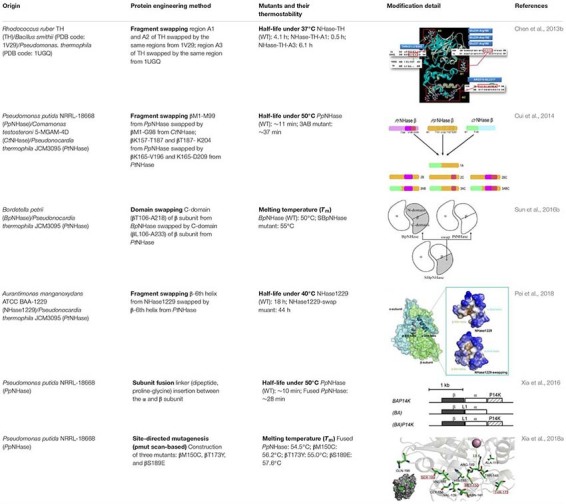

It is reported that the thermostability of NHase from *P. putida* could be obviously improved by the fusion with EAK16 and ELK16, which known as self-assembling amphipathic peptides. Fusing these peptides with the NHase induced the formation of active inclusion body and the half-life of this enzyme could also be enhanced in a large extent compared with the wild-type NHase (Liu et al., 2014b).

Most recently, inspired by the gene structure of eukaryotic NHase of which the separated subunits are presented as one peptide, Xia et al. (2016) constructed a new type of prokaryotic NHase with only one polypeptide by fusing the β- and α-subunits of the NHase from *P. putida* NRRL-18668 (Table 3). It is known that fusion strategy is able to help simplify the topologies of protein complex and therefore optimize protein assembly (Marsh et al., 2013). Furthermore, it is believed that by gene fusion, protein stability can be enhanced (Grossmann et al., 1997; Legardinier et al., 2008; Azzam et al., 2012). In this case, the fusion NHase exhibited excellent thermostability and became more tolerant to high concentration of amide products compared with the wild-type NHase. A follow-up research that merged the advantages of the pmut scan application of Rosetta 3.4 (Kuhlman et al., 2003; Leaver-Fay et al., 2011) and MD simulations was used to further improve the thermostability of the subunit-fused NHase by the same group (Table 3; Xia et al., 2018a). Calculation of the folding free energy ΔΔ*G* by Rosetta provided several single point mutation candidates and with the help of MD simulation, three best mutants with enhanced thermostability were obtained.

## Applications of NHase

It is well known that the industrialization of NHase in the field of acrylamide biosynthesis is one of the most successful and representative examples in industrial biotechnology. There are several major manufacturers dedicated the enzymatic hydration approach for acrylamide production. Using NHase as catalyst, Mitsubishi Corporation could produce more than 200,000 tons of acrylamide from acrylonitrile per year (Sakashita et al., 2008). BASF in Germany and Lonza in Switzerland also show strong competitiveness in the production of nicotinamide (Chuck, 2005). Besides acrylamide production, NHases have been used as industrial biocatalysts in the commercial production of nicotinamide and 5-cyanovaleramide. Recently, the potential applications of NHase for other valuable amides synthesis has been disclosed. Zhao et al. (2019) found that NHase from a N_2_-fixing bacterium *Ensifer meliloti* could biodegrade indole-3-acetonitrile to indole-3-acetamide, which acts as an important building block of the auxin-class plant hormone. NHase from *Rhodococcus pyridinivorans* NIT-36 was found to be able to produce lactamide, an industrially important lactic amide which is widely used in the cosmetic industry, with high catalytic activity (Singh et al., 2019). 2,6-Difluorobenzamide, an important intermediate in pesticide industries, could be synthesized by NHase from *A. manganoxydans* ATCC BAA-1229 with a final concentration equals to 314 g/L through a simple batch process (Yang et al., 2019b).

Due to the relative instability of NHase, most of the corresponding industrial catalysis applications are carried out using whole cells. However, some industrially important nitriles cannot readily permeate the cell membrane, causing less substrate to be hydrated. Furthermore, the conventional problems of maintaining the enzyme activity throughout the repeated use of free resting cells, as well as preservation also remains in the case of the biotransformation of nitriles. Recent progress in enzyme immobilization methods has provided an alternative method to improve the stability and reusability of NHase. Gao et al. (2014) immobilized NHase ES-NHT-118 in mesoporous onion-like silica and the adsorbed NHase was then cross-linked with dextran polyaldehyde. Such a complex proved to be effective in helping simplify the amide production technology and maintain NHase activity (Gao et al., 2014). By using polyurethane foam cubes as immobilization matrix, growing cells of *Rhodococcus* UKMP-5M showed the best nitrile-hydrolyzing enzyme activity and were found to tolerate higher concentrations of acrylonitrile (Sjahrir et al., 2016). More recently, NHase was immobilized in the dopamine functionalized SBA-15. The immobilized NHase showed improved thermal, pH and storage stability without huge losses in catalytic efficiency (Chen et al., 2017). Besides, biological metal-organic frameworks (BioMOFs) were recently found to be ideal candidates for NHase immobilization (Wang et al., 2020). Pei et al. (2020) encapsulated a NHase from *A. manganoxydans* into the cobalt-based mesoporous MOFs. The biomimetic mineralized NHase showed high catalytic activity toward 3-cyanopyridine and remained 40% of the maximum activity at 70°C (Pei et al., 2020). All of these advances in NHase immobilization might gain popularity in industrial settings in the near future.

Nitrile hydratase not only are used for the production of amide intermediates, but also proven to be useful in environmental protection. The main applications include biodegradation of acetonitrile, adiponitrile, acetamiprid, and 2-amino-2,3-dimethylbutyronitrile. With the help of NHase, biocatalytic nitrile hydrolysis represents a successful strategy to remove highly toxic nitriles from industrial waste (Feng et al., 2008). Guo et al. (2019) reported that a novel NHase from *Streptomyces canus* CGMCC 13662 was involved in the biodegradation of the neonicotinoid insecticide acetamiprid. The NHase shows high thermostability and was found to be acid-alkaline tolerant (Guo et al., 2019).

## Concluding Remarks and Future Perspectives

Researchers from all over the world have dedicated themselves to the development of NHase with the aim of advancing NHase research into a new era. Various NHases have been isolated, well characterized and engineered. Great progress has been made on clarifying the catalytic mechanisms of these enzymes. We have observed the promise of the industrial application of NHase not only in traditional amide production, but also in environmental and other fields. However, it has not been a simple process, and much work remains to be accomplished. Limited acceptability of substrates, low tolerance to substrate or product, and instability under relatively high temperature still hinder the practical applications of NHase. Potentially, with the rapid advances in fields such as gene mining, protein engineering, synthetic biology, and bioinformatics, the current issues facing NHase could be addressed and the applications of this green catalyst could be elevated to new heights.

## Author Contributions

All authors listed have made a substantial, direct and intellectual contribution to the work, and approved it for publication.

## Conflict of Interest

The authors declare that the research was conducted in the absence of any commercial or financial relationships that could be construed as a potential conflict of interest.
